# Association of Innate and Acquired Aerobic Capacity With Resilience in Healthy Adults: Protocol for a Randomized Controlled Trial of an 8-Week Web-Based Physical Exercise Intervention

**DOI:** 10.2196/29712

**Published:** 2021-11-29

**Authors:** David T Ochmann, Keito F A Philippi, Peter Zeier, Magdalena Sandner, Barlo Hillen, Elmo W I Neuberger, Inigo Ruiz de Azua, Klaus Lieb, Michèle Wessa, Beat Lutz, Perikles Simon, Alexandra Brahmer

**Affiliations:** 1 Sports Medicine, Disease Prevention and Rehabilitation Johannes Gutenberg-University Mainz Mainz Germany; 2 Clinical Psychology and Neuropsychology, Institute for Psychology Johannes Gutenberg-University Mainz Mainz Germany; 3 Institute of Physiological Chemistry University Medical Center of the Johannes Gutenberg-University Mainz Mainz Germany; 4 Department of Psychiatry and Psychotherapy University Medical Center of the Johannes Gutenberg-University Mainz Mainz Germany; 5 Leibniz Institute for Resilience Research Mainz Germany; 6 Extracellular Vesicles Research Group, Institute of Developmental Biology and Neurobiology Johannes Gutenberg-University Mainz Mainz Germany

**Keywords:** stress resilience, eHealth, aerobic capacity, peak oxygen uptake, cortisol, kynurenic acid, endocannabinoids

## Abstract

**Background:**

Physical activity alleviates chronic stress. The latest research suggests a relationship between resilience and physical fitness. Beneficial adaptations of the hypothalamic-pituitary-adrenal axis, sympathetic nervous system, endocannabinoid system, and tryptophan pathway, which are induced by an active lifestyle, are considered to be conducive to resilience. However, detailed knowledge on the molecular link between the effects of acute and chronic physical exercise and improved resilience to stress in humans is missing. Moreover, the relationship between innate and acquired aerobic capacity and resilience is poorly understood.

**Objective:**

The aim of this study is to implement a human exercise intervention trial addressing the following main hypotheses: a high innate aerobic capacity is associated with high resilience to stress, and web-based physical exercise training improves aerobic capacity of physically inactive adults, which is accompanied by improved resilience. In this setting, we will analyze the relationship between resilience parameters and innate and acquired aerobic capacity as well as circulating signaling molecules.

**Methods:**

A total of 70 healthy, physically inactive (<150 minutes/week of physical activity) adults (aged 18-45 years) will be randomly assigned to an intervention or control group. Participants in the intervention group will receive weekly training using progressive endurance and interval running adapted individually to their remotely supervised home training performance via web-based coach support. A standardized incremental treadmill exercise test will be performed before and after the intervention period of 8 weeks to determine the innate and acquired aerobic capacity (peak oxygen uptake). Before and after the intervention, psychological tests and questionnaires that characterize parameters implicated in resilience will be applied. Blood and saliva will be sampled for the analysis of cortisol, lactate, endocannabinoids, catecholamines, kynurenic acid, and further circulating signal transducers. Statistical analysis will provide comprehensive knowledge on the relationship between aerobic capacity and resilience, as well as the capacity of peripheral factors to mediate the promoting effects of exercise on resilience.

**Results:**

The study was registered in October 2019, and enrollment began in September 2019. Of the 161 participants who were initially screened via a telephone survey, 43 (26.7%) fulfilled the inclusion criteria and were included in the study. Among the 55% (17/31) of participants in the intervention group and 45% (14/31) of participants in the control group who completed the study, no serious adverse incidents were reported. Of 43 participants, 4 (9%) withdrew during the program (for individual reasons) and 8 (19%) have not yet participated in the program; moreover, further study recruitment was paused for an indeterminate amount of time because of the COVID-19 pandemic.

**Conclusions:**

Our study aims to further define the physiological characteristics of human resilience, and it may offer novel approaches for the prevention and therapy of mental disorders via an exercise prescription.

**International Registered Report Identifier (IRRID):**

DERR1-10.2196/29712

## Introduction

### Background

Physical inactivity is one of the major risk factors for global mortality; therefore, an active lifestyle is highly important to promote health and longevity. The risk for cardiovascular diseases, type 2 diabetes, osteoporosis, metabolic syndrome, specific cancers, and several other diseases is reduced by being physically active [1], and physical exercise can be prescribed as a medicine for 26 common chronic diseases [2]. In addition, mental health is positively affected by regular physical activity. Physical exercise induces the preservation of brain volume, which is associated with cognitive benefits [3,4]; it promotes blood-brain barrier integrity and protects against central nervous system infiltration of immune cells [5], and neurodegenerative diseases, such as Alzheimer and Parkinson disease, are counteracted by an active lifestyle [6].

To show the influence of exercise type, frequency, duration, and intensity on mental health, a cross-sectional study was conducted with 1.2 million individuals in the United States between 2011 and 2015 [7]. It was shown that regularly physically active persons have approximately half as many days of poor mental health than physically inactive persons. Furthermore, it was demonstrated that any kind of exercise had a reduced mental health burden compared with not exercising. A duration of at least 45 minutes with a frequency of 3 to 5 times a week is recommended. However, the authors also cautioned that more exercise is not always better [7].

To improve peak oxygen uptake (VO_2_peak), it has been shown that individuals who perform interval training have a higher increase in VO_2_peak [8] or have a shorter exercise duration with similar increases in VO_2_peak compared with those who perform continuous exercise [9-11]. The comparison between low- and high-fit groups showed that the high-fit participants reacted and recovered faster within and after a psychological stress situation [12,13]. Furthermore, high-fit individuals had a lower cortisol concentration at rest and higher variability during the stressor compared with low-fit individuals. Moreover, a low cardiorespiratory fitness at the age of 18 years is associated with serious depression in later life [14], whereas implementing regular physical exercise training induces comparable efficacy in the treatment of major depression as antidepressant medication [15]. The described positive effects of physical exercise on mental health are accompanied by increased overall resilience [16].

Currently, there is no uniform and stringent definition of resilience [17-19]. A common psychological definition of resilience is “a person’s ability to adapt successfully to acute stress, trauma or more chronic forms of adversity” [20]. According to Kalisch et al [17], it is defined as the “empirically observable phenomenon [...] that someone does not develop lasting mental health problems although he or she is subject to adversity.” Recent resilience theories suggest a distinction to be made between resilience as an outcome, that is, the change in mental health relative to the stressor burden in a certain period, and resilience mechanisms, that is, variables that directly affect resilient outcomes [17]. By now, several psychosocial characteristics of a resilient phenotype (eg, lower levels of denial, avoidant coping behavior, and high positive emotionality) and multiple ways of promoting it (eg, improving problem-solving and planning skills) have been described [16]. Emotion regulation ability is considered a potential resilience mechanism and finds prominence in dynamic resilience theories [17,21]. Previous cross-sectional studies have indicated a positive relationship between physical activity and emotion regulation [22,23]. However, the driving neural circuits and underlying molecular pathways in resilience, especially in the context of physical exercise, are only beginning to be unraveled [16].

The major neuroendocrine and neural drivers of the responses to stress are the hypothalamic-pituitary-adrenal (HPA) axis, which mediates glucocorticoid (cortisol) release, and the sympathetic nervous system, which initiates the release of catecholamines (epinephrine and norepinephrine) in interaction with the immune system. Adequate activation of these systems in response to psychological or physical stress and subsequent restoration of homeostasis is critical to physical and mental health [16,24]. Malfunction of this responsive orchestration is associated with the development of several chronic diseases, including psychiatric diseases, autoimmune diseases, and cardiovascular diseases, contributing to mortality worldwide [25-27]. Acute bouts of physical exercise activate the HPA axis and the sympathetic nervous system, leading to increased stress hormone release followed by immune cell mobilization in a dose-responsive fashion [28-30]. When cessation of the physical stressor and the ensuing reconstitution of a homeostatic state occurs after a moderate period, adaptive mechanisms may result in improved stress reactivity. These adaptations include neurogenesis, improved synaptic plasticity and neuroprotection (eg, by increased levels of brain-derived neurotrophic factor [30,31]), and the promotion of an anti-inflammatory state (eg, by enhanced anti-inflammatory cytokines and reduced c‑reactive protein release [32]). Consequently, regular physical exercise may increase resilience by promoting a more adaptive activation of the HPA axis. Similarly, an exercise-induced shift in the tryptophan pathway, resulting in decreased kynurenine and increased kynurenic acid plasma levels, is considered to promote neuroprotection and resilience [33,34]. Furthermore, physical exercise activates the endocannabinoid system, which is considered to result in cognitive benefits and an antidepressant effect [35-37]. Thus, exercise prescription constitutes a useful means for the prevention and treatment of psychological and neurodegenerative diseases and moreover offers the possibility to enhance resilience in the general population.

Structured physical exercise interventions have been successfully implemented to improve disease states in brain diseases, such as major depression, Alzheimer disease, and Parkinson disease [15,38,39]. However, personalized training in a face-to-face fashion requires a high workload for coaches, and participants need to adhere to a strict time schedule. Individualized web-based physical exercise interventions allow trainers to remotely supervise multiple participants in a time- and cost-effective manner, for example, by providing weekly feedback. In turn, participants are highly flexible when integrating the training sessions into their daily routines and still receive individualized training programs from a coach. Our research group had previously shown in different types of patients that a web-based exercise program is useful, does not harm any participant, and improves physiological parameters, such as VO_2_peak [40,41], and clinically relevant parameters [42-45].

Physical activity is positively associated with blunted stress reactivity and shorter stress recovery in response to various stressors [46,47]. To investigate acute stress reactivity and recovery in a psychophysiological laboratory, an experimental paradigm that significantly induces stress at the psychological and physiological levels is needed. Dickerson and Kemeny [48] identified several elements that reliably elicit significant HPA axis responses in participants: (1) physical stressors (eg, heat or cold), (2) mental challenges (eg, arithmetic tasks and working memory tasks), and (3) social evaluation (eg, giving a speech in front of a jury). In psychological stress research, there are several established stress paradigms that make use of these elements. The Socially Evaluated Cold Pressor Task (SECPT) [49] and the ScanSTRESS-C [50] for acute stress induction are experimental paradigms that have previously been proven effective in eliciting acute stress responses at the psychological (ie, decreases in well-being), endocrine (ie, high levels of catecholamines and cortisol), and physiological (ie, higher heart rate levels) levels [50-52].

### Objectives

In this study, we implement an 8-week randomized controlled trial of a web-based, individualized physical exercise training intervention to evaluate the link between aerobic capacity and resilience in healthy untrained adults. Therefore, baseline values of performance diagnostics to determine the innate aerobic capacity (VO_2_peak) as well as emotion regulation abilities, stress reactivity, and stress recovery (saliva cortisol) as potential resilience mechanisms are estimated. We hypothesize that (1) participants with a high innate aerobic capacity will show a higher resilience to stress compared with participants with low aerobic capacity. This will be followed by 8 weeks of individualized web-based physical exercise training combining continuous and interval-type running exercises with the aim of progressively increasing aerobic capacity. After completion of the exercise intervention, performance diagnostics will be repeated to estimate the acquired aerobic capacity, as well as to assess the psychological parameter. In addition, secondary outcomes of molecular factors, including hair cortisol levels, endocannabinoids, catecholamines, cytokines, and cell-free DNA, and results of several questionnaires will be determined throughout the study. We hypothesize that (2) the exercise intervention will improve the aerobic capacity of the intervention group, which (3) will be accompanied by improvements in resilience factors. This will offer the possibility to exploratively study the underlying molecular mechanism (stress hormone release and immune reactions) in the promotion of resilience by regular physical activity.

## Methods

### Trial Design and Participants

**Figure 1:**
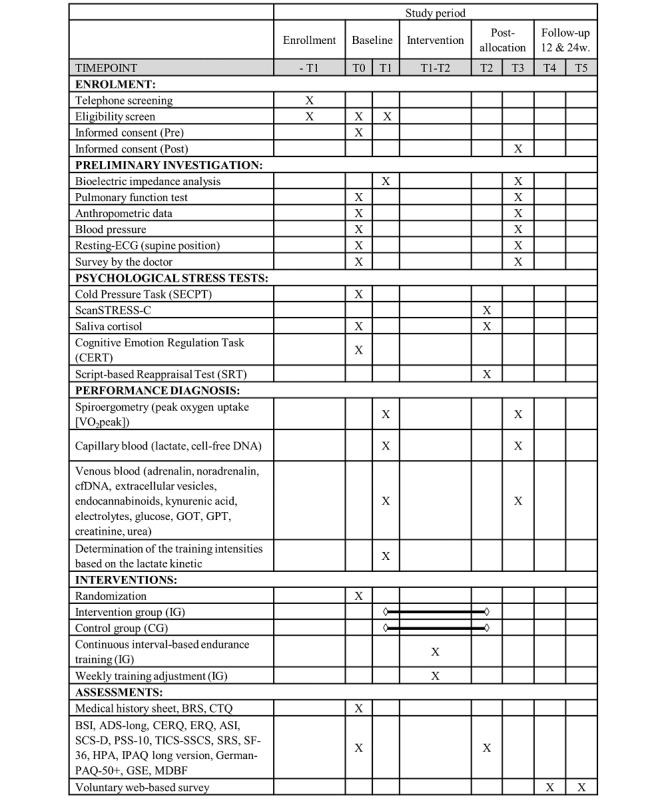
SPIRIT (Standard Protocol Items: Recommendations for Interventional Trials) diagram of enrollment, interventions, and assessments. ADS: Allgemeine Depressionsskala; ASI: Anxiety Sensitivity Index; BRS: Brief Resilience Scale; BSI: Brief Symptom Index; CERQ: Cognitive Emotion Regulation Questionnaire; CG: Control Group; CTQ: Childhood Trauma Questionnaire; ECG: electrocardiogram; ERQ: Emotion Regulation Questionnaire; GOT: Glutamate Oxalacetate Transaminase; GPT: Glutamate Pyruvate Transaminase; GSE: General Self-Efficacy Scale; HPA: Habitual Physical Activity Questionnaire; IG: Intervention Group; IPAQ: International Physical Activity Questionnaire; MDBF: Mehrdimensionaler Befindlichkeitsfragebogen; PAR-Q: Physical Activity Readiness Questionnaire; PSS-10: Perceived Stress Scale–10; SCS-D: Self-compassion Scale–German Version; SECPT: socially evaluated cold pressor test; SF-36: Short-Form Health Survey; SRS: Stress Reactivity Scale; TICS-SSCS: Trier Inventory for the assessment of Chronic Stress–Screening Scale For Chronic Stress.

This study is designed as a prospective randomized controlled trial with 2 phases of data collection before (baseline examination; time point 0 [T0] and time point 1 [T1]) and after (final examination; time point 2 [T2] and time point 3 [T3]) 8 weeks of web-supervised physical exercise training intervention (T1-T2; Figure 1). The multidisciplinary single-center trial is a collaboration among the Institute of Physiological Chemistry, Department of Psychiatry and Psychotherapy, Department of Clinical Psychology and Neuropsychology, and Department of Sports Medicine, Disease Prevention and Rehabilitation. The medical association Rhineland-Palatinate approved the study (July 29, 2019, ID: 2019-14305), and the study was registered with the German Clinical Trials Register (DRKS; DRKS00018078; October 2, 2019) and conformed to the standards of the Declaration of Helsinki of the World Medical Association. Administrative changes of the protocol, if needed, are minor corrections and clarifications that have no effect on the way the study is to be conducted. An amendment will be approved by the Ethics Committee Landesärztekammer Rhineland-Palatinate before implementation. This protocol follows the guidelines from the SPIRIT (Standard Protocol Items: Recommendations for Interventional Trials) [53] and the CONSORT-eHEALTH checklist (Multimedia Appendix 1) [54]. A SPIRIT diagram of enrollment, interventions, and assessments is provided in Figure 1.

The primary (VO_2_peak and saliva cortisol) and key secondary outcomes, trial methods, and designs are summarized in the World Health Organization trial registration data set (Textbox 1). This study includes 4 time points of investigation of physiological and psychological parameters, including the primary end points of a change in VO_2_peak and saliva cortisol from the baseline: at T0 and T1, baseline values will be estimated, and at T2 and T3, data collection following the control or exercise intervention will be performed. T0 and T2, as well as T1 and T3, are structured in the same way, except for the separately listed psychological examinations at the measurement points. T1 and T3 will be performed after a maximum of 10 days after T0 and T2, respectively. Written consent after a detailed oral explanation will be obtained from all participants at the beginning and after completion of the study by the responsible study physician. At the beginning of the study (T0), the participants will not be fully informed about the purpose and aim of the psychological stress tests. Full clarification will take place at T3. If a participant wishes to discontinue the study prematurely, they will be fully informed about the purpose and aim of the psychological stress tests.

**Figure 2:**
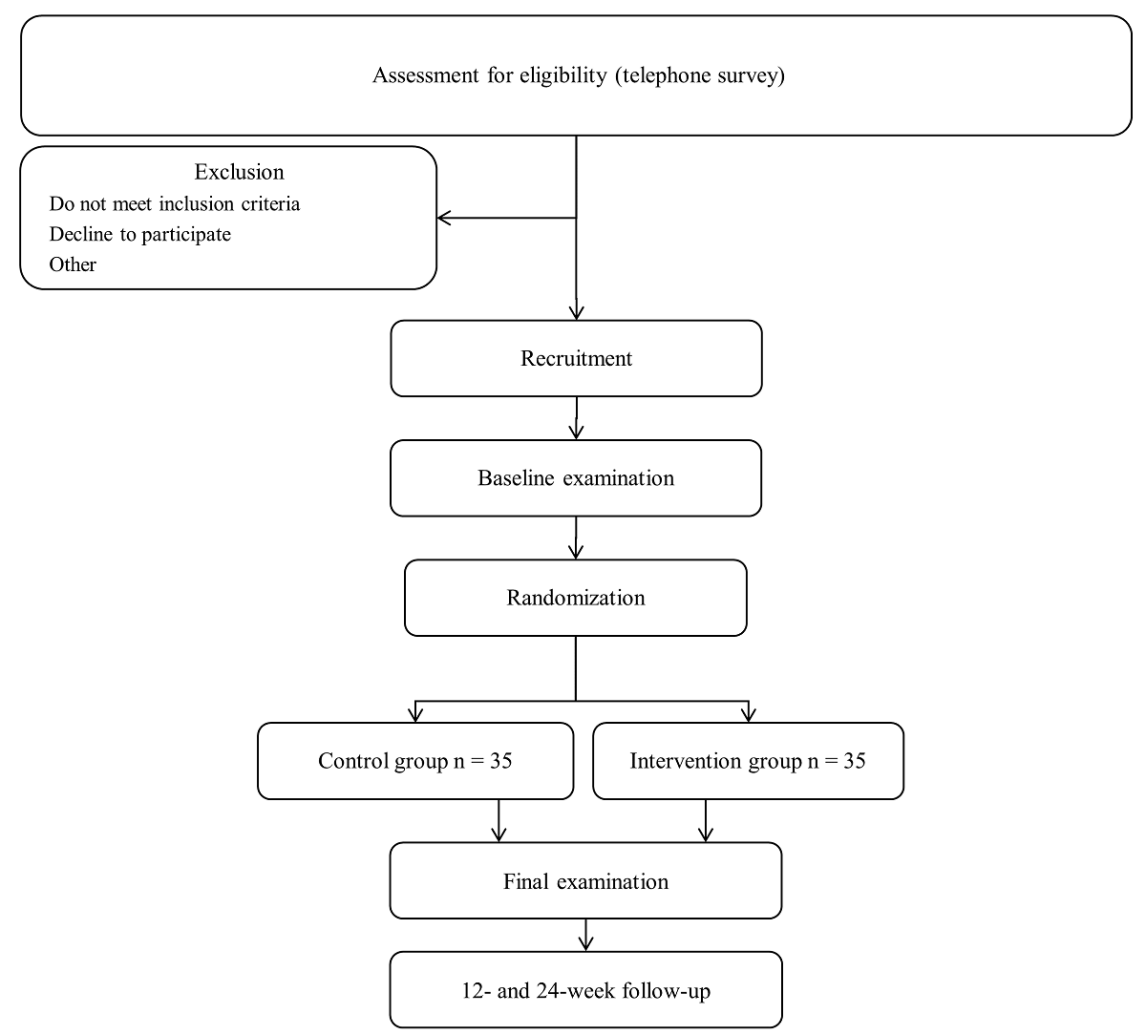
Study design flow diagram.

Participant recruitment commenced in September 2019. Participants were and will be recruited via flyer announcements for the study (Multimedia Appendix 2) at multiple locations at the Johannes Gutenberg-University of Mainz and the University Medical Center campuses, as well as diverse places for leisure activities, as impersonal recruitment. Inclusion into the study is a two-step procedure beginning with a telephone survey according to the eligibility criteria (Textbox 2). At the first visit, sport capability will be confirmed and the participants will be included in the study. Blinding of participants will not take place. After telephone screening and recruitment, participants will be randomized (Figure 2) into the intervention group (IG) or control group (CG). The IG will participate in the 8-week sports intervention based on a combination of continuous and interval-type running exercises. Participants in the IG will be told not to engage in further high-intensity sports activities. Participants in the CG will be told not to change their daily lifestyles during the intervention period.

**World Health Organization trial registration data set.**

**Trial registration data set**

- Primary registry and trial identifying number: German Clinical Trials Register DRKS00018078
- Date of registration in primary registry: October 2, 2019
- Secondary identifying numbers: none
- Source of monetary or material support: Boehringer Ingelheim Foundation
- Primary sponsor: Boehringer Ingelheim Foundation
- Secondary sponsor(s): not applicable
- Contact of public queries: ochmann@uni-mainz.de (DO); albrahme@uni-mainz.de (AB)
- Contact for scientific queries: albrahme@uni-mainz.de (AB)
- Public title: mediators of a link between innate or acquired aerobic capacity and stress resilience
- Scientific title: The association of innate and acquired aerobic capacity with resilience in healthy adults: Protocol for an 8-week randomized controlled, web-based, physical exercise intervention study
- Countries of recruitment: Germany
- Health condition(s) or problem(s) studied: healthy participants
- Intervention(s): web-based exercise intervention; control
- Key inclusion criteria:
  Age: 18-45 years
  Healthy
  Suitable for sports
  Physically inactive (<150 minutes/week physically active)
  Gender: male and female
- Key exclusion criteria:
  Absolute contraindications to exercise
  Smoker or other tobacco consumption
  High blood pressure
  Long-term medication intake
  Hairless participants (<1 cm)
  Shift worker
  Participation in another study
  Psychological disorders
- Study type:
  Interventional
  Allocation: randomized, controlled
  Intervention model: parallel assignment
  Primary purpose: prevention
- Date of first enrollment: September 10, 2019
- Target sample size: 70
- Recruitment status: Recruiting
- Primary outcome(s):
  Improvement of the maximum oxygen uptake through an 8-week web-based training intervention.
  Investigation of the link between the changes in maximum oxygen uptake induced during training intervention and the associated changes in salivary cortisol induced during a stress test.
- Key secondary outcomes:
  Investigation of the link between maximum oxygen uptake and resilience by determining physiological and biochemical markers in the untrained state before an 8-week web-based training intervention.
  Investigation of the link between the changes in maximum oxygen uptake and resilience induced through training intervention by determining physiological and biochemical markers.
  Sustainability of the change in resilience 12 and 24 weeks after 8 weeks of web-based training intervention.

### Randomization

The included participants will be randomly assigned with a 50%-50% allocation to the IG or CG. Randomization will be performed using the EXCEPT NUMBER command in Microsoft Excel. In addition, randomization will be stratified by gender with an expected ratio of 50%-50% and BMI with an expected ratio of 33% overweight (BMI ≥25) and 67% normal weight (BMI <25) to recruit CG and IG participants with homogeneous baseline characteristics.

### Eligibility Criteria

To work with a homogenous study population and to reduce the influence of different characteristics of the study population on study outcomes, the inclusion and exclusion criteria for participating in the study were chosen as listed in Textbox 2. It is known that both neural and behavioral processing of stress is altered in clinical samples with conditions such as depression [55] or anxiety disorders [56]. To minimize differences between participants, we excluded individuals with current or past psychological treatment as assessed in our standardized telephone screening. To reduce the impact of hormonal contraceptives in women on stress reactivity (saliva cortisol) and emotional memory [57], only women taking no hormonal contraceptives are included in our study. Alcohol abuse was defined according to the *Diagnostic and Statistical Manual of Mental Disorders* (Fourth Edition) [58] as a regular or daily drinking routine.

**Inclusion and exclusion criteria.**

**Inclusion criteria**

- Age: 18-45 years
- Nonsmokers
- Normal blood pressure (≤140/90 mm Hg)
- Suitable for sports
- Voluntary participation
- No medication
- Physically inactive (<150 minutes/week physically active)
- Time for 8-week intervention
- Signing the consent form
- Gender: male and female

**Exclusion criteria**

- Age: <18 or >45 years
- Smoker or other tobacco consumption
- High blood pressure
- Not suitable for sports
- Constrained participation
- Long-term medication intake
- Trained (>150 minutes/week physically active)
- Probably no time for intervention
- Rejection by the participant
- Lack of ability to consent or doubts about the ability to consent
- Hairless participants (<1 cm)
- Shift worker
- Clinically significant 12-channel electrocardiogram abnormality determined by the physician
- Alcohol or drug abuse within the past year before screening, positive urine drug test, or positive alcohol test during screening
- Infection with HIV, Hepatitis C virus, or Hepatitis B virus
- Participation in another study
- Other unspecified reasons that, from the investigator's point of view, militate against participation in the study
- Cardiovascular, metabolic, pulmonary, and muscular diseases after the *International Classification of Diseases, Tenth Revision*, acute or anamnestic
- Psychological disorders diagnosed according to *International Classification of Diseases, Tenth Revision* or *Diagnostic and Statistical Manual of Mental Disorders* (Fourth Edition), acute or anamnestic (last 24 months)
- Female participants taking hormonal contraceptives

### Preliminary Investigation (T0/T1 and T2/T3)

The first visit (T0) includes the initial screening of the participant’s height and weight. A pulmonary function test will be used to determine vital capacity, forced expiratory volume in 1 second (Spirometry, Bodybox 5500, Medisoft Group) and, if applicable, to identify the presence of undiagnosed restrictive or obstructive respiratory abnormalities [59]. Furthermore, the cardiological values of resting blood pressure and resting electrocardiogram (Schiller AT-60) will be measured in a supine position to ensure proper cardiovascular function. To determine the long-term cortisol value over 2 months, a 2 cm–long strand of hair will be cut off at the back of the head directly from the skin [60]. A bioelectric impedance analysis (In Body 3.0, Biospace) and blood counts will be conducted during the second visit (T1) in the morning before the incremental running test after a minimum of 8 hours of overnight fasting. After the completion of the control or exercise intervention, the described parameters will be reassessed during the third or fourth visit (T2 or T3).

### Questionnaires (T0 and T2)

Owing to the multidimensional approach to stress resilience outlined above, we required a lot of information about the participants’ lifestyle as well as psychological and medical anamnesis with the help of several questionnaires. To minimize the total experimental time, participants will complete part of the questionnaires on the web before their arrival at T0. Further questionnaires will be completed within the resting and recovery periods during the on-site appointments.

#### Psychological Questionnaires

##### Brief Resilience Scale (Only T0)

The Brief Resilience Scale is a 6-item questionnaire that considers the dimension *bounce back* from stress on a 5-point Likert scale. The scale exhibits good psychometric properties with high internal consistency and retest reliability [61]. The German version of the Brief Resilience Scale was validated in 2018 [62].

##### Childhood Trauma Screener (Only T0)

The Childhood Trauma Screener (CTS) is a 5-item brief instrument for the assessment of childhood abuse and neglect on a 5-point Likert scale [63]. The CTS is based on the Childhood Trauma Questionnaire, which includes 28 items on physical, emotional, and sexual abuse as well as emotional and physical neglect [64,65]. These questions of the CTS deal with experiences during childhood and youth. The CTS showed a high correlation (*r*=.88; *P*<.001) with the total Childhood Trauma Questionnaire score. The internal consistency was .757 (Cronbach α; n=499).

##### Brief Symptom Index-18

The Brief Symptom Index-18 is an 18-item scale, which is divided into 3 syndromes—somatization, depression, and anxiety—with 6 items each on a 5-point Likert scale [66]. The German version of the Brief Symptom Index-18 was validated in 2011. It showed a satisfactory-to-high consistency of the different syndromes (Cronbach α between .63 and .93) [67].

##### General Depression Scale (Long Version)

The General Depression Scale (German translation: *Allgemeine Depressionsskala* [ADS]) is the most common German version of the Center for Epidemiologic Studies Depression Scale [68]. The first test publication of ADS was in 1993 [69]. The second edition of the manual with new samples and standard data was published in 2012 [70]. No changes were made to the items. Only the critical threshold value of the long version (ADS-L) for the screening of depression was corrected from >23 to >22 [70]. With its 20 items, the ADS-L considers affective, cognitive, somatic, and social depression symptoms, among others. The answers are given on a 4-point scale from 0 (rarely or not at all [<1 day]) to 3 (mostly all the time [5-7 days]). The internal consistency (Cronbach α) of the ADS-L in adults in multiple population samples is between .85 and .92, and the test-retest reliability (2-8 weeks) is .51-.67.

##### Cognitive Emotion Regulation Questionnaire

The Cognitive Emotion Regulation Questionnaire covers 9 strategies (self-blame, blaming others, acceptance, refocusing on planning, positive refocusing, rumination or focus on thought, positive reappraisal, putting into perspective, and catastrophizing) and 36 items of cognitive emotion regulation with a range from 1 ([almost] never) to 5 ([almost] always) [71]. The German version differs in the item number. Here the questionnaire was shortened to 27 items (3 items per dimension) [72,73]. A good psychometric quality (factorial validity and acceptable-to-good reliability [.70<α<.84]) could be shown in a clinical sample [72].

##### Emotion Regulation Questionnaire

The Emotion Regulation Questionnaire deals with the most common preferences of 2 applied strategies for emotion regulation: suppression (4 items) and reappraisal (6 items) [74]. The German version was first published in 2009 [75]. On a 7-point Likert scale from 1 to 7 (1=not correct at all; 4=neutral; and 7=completely correct), the participants can choose a number. Internal consistency (Cronbach α) for reappraisal was .79, and for suppression, .73 [74]. The test-retest reliability across 3 months was .69 for both strategies [74].

##### Anxiety Sensitivity Index-3

Anxiety Sensitivity Index-3 is an 18-item scale with 3 factors (physical, cognitive, and social concerns) on a 5-point Likert scale [76]. The German version was first published in 2009. The internal consistency is between .86 and .89 [77]. Satisfactory measurement accuracy and good validity can be seen in comparison with the English version [77].

##### Self-compassion Scale (German Version)

The Self-compassion Scale (SCS) contains 26 items with originally 3 basic components: (1) self-kindness, (2) common humanity, and (3) mindfulness, which are answered on a 5-level scale (1=very rarely and 5=very often) [78]. The German version of the SCS (SCS-D) was first checked for reliability and validity in 2011 [79]. In comparison with the original, a 6-factorial structure (self-kindness, self-condemnation, common humanity, isolation, overidentification, and mindfulness) was found and as expected, correlates with subjective well-being and psychological strain. With the German version of the SCS, an economic, reliable, and valid assessment of self-compassion is available.

##### Perceived Stress Scale-10

The Perceived Stress Scale (PSS-14) contains 14 items, which are answered on a 5-point response scale (0=never and 4=very often) [80]. On the basis of principal component analysis, a low factor loading of 4 items was determined and then dropped. The shortened PSS-10 shows a slightly increased reliability (Cronbach α=.78 vs Cronbach α=.75) and a similar validity [81]. The German version of the PSS-10 shows good internal consistency (Cronbach α=.84) and construct validity between perceived stress and depression, anxiety, fatigue, procrastination, and quality of life [82,83]. PSS-10 is an economic, reliable, and valid assessment tool for assessing perceived stress.

##### Trier Inventory for the Assessment of Chronic Stress–Screening Scale for Chronic Stress

The TICS-SSCS (Trier Inventory for the assessment of Chronic Stress–Screening Scale for Chronic Stress) comprises 12 items [84]. The TICS-SSCS measures the frequency of self-perceived overall stress in 5 different stress domains in the past 3 months: chronic worrying, work-related overload, social overload, excessive demands, and lack of social recognition. The frequency of stress in the 5 stress domains is recorded with the values *never* (0 points), *rarely* (1 point), *sometimes* (2 points), *frequently* (3 points), and *very frequently* (4 points), and in the end, an average score can be calculated. The internal consistency for the TICS-SSCS is rated as very good, with Cronbach α=.91 [84,85].

##### The Stress Reactivity Scale

The 29-item Stress Reactivity Scale measures the duration and magnitude of an effective response that a person displays in various stress situations. Each item consists mainly of 2 parts: (1) a typical stress situation and (2) 3 response items. From this, the total score can be calculated. A satisfying Cronbach α between .71 and .82 for the various subscales and good retest reliability coefficients over 7 months between .63 and .84 could be shown [86].

##### General Self-efficacy Scale

The General Self-efficacy Scale is a 10-item psychometric scale that assesses general optimistic self-conviction [87]. The items are answered on a 4-level scale (1=not correct; 2=hardly correct; 3=rather correct; and 4=exactly correct). At the end, all points are summed up, and the result is a score between 10 and 40. The internal consistency of Cronbach α is between .75 and .91. Confirmatory factor analysis could confirm the single-factor structure of the scale. The scale shows a good retest reliability of .67 [88].

##### Multidimensional Mood State Questionnaire

To assess psychological responses to acute laboratory stress induction, we will use the Multidimensional Mood State Questionnaire (German translation: *Mehrdimensionaler Befindlichkeitsfragebogen* [MDBF]) [89]. The MDBF consists of 24 adjectives that reflect positive or negative emotional states. Participants’ ratings on a 5-point Likert scale could be summed up to a total score of subjective well-being, where higher scores reflect a more positive emotional state. Sufficient reliability (Cronbach α=.80-.92) and validity of the MDBF have been confirmed in several studies [90-92]. As in previous studies by our group [50,93], we will use the MDBF at multiple time points before and after the stress tasks at T0 and T2 to assess variations in psychological well-being in response to stress (–2 minutes, +15 minutes, and +60 minutes relative to stress onset).

#### Sports Medicine Questionnaires

##### Habitual Physical Activity Questionnaire

The Habitual Physical Activity Questionnaire is divided into 3 indices evaluating (1) occupational physical activity, (2) sports, and (3) leisure time, and it originally included 16 items [94]. The German version of the Habitual Physical Activity Questionnaire was compared with the original version in 2001 and was reduced by 2 items after item-analytical analysis [95]. The test-retest reliability of the indices of physical activity over 3 months is .80 and .90 for (1) and (2) and .74 for (3) [94].

##### International Physical Activity Questionnaire (Long Version)

The long version of the International Physical Activity Questionnaire contains 27 items and collects physical activity values in different domains (occupational, transport, yard or garden, household, leisure, and sitting) and different intensities (moderate and vigorous) within the past 7 days [96]. The German long version was validated on German adolescents [97]. It is an acceptable, valid, and reliable questionnaire for assessing physical activity in many countries [96].

##### Short-Form Health Survey

The 36-item Short-Form Health Survey is a cross-disease assessment instrument for measuring the health-related quality of life of the general population and patients [98]. It consists of 8 dimensions of subjective health: physical functioning (10 questions), social functioning (2 questions), role limitations (physical problems, 4 questions; emotional problems, 3 questions), general mental health (psychological distress and well-being; 5 questions), vitality (energy and fatigue; 4 questions), bodily pain (2 questions), and general health perception (5 questions). In addition to the 8 dimensions, one question is used to request health changes [99]. These dimensions can be assigned to the functional status and well-being of the basic dimensions. The version of the Short-Form Health Survey-36 used here refers to the past 4 weeks. Except for social functioning (Cronbach α=.73), the 7 other dimensions showed acceptable internal consistency, with a Cronbach α≥.85. The test-retest reliability is excellent within 2 weeks [99].

##### Physical Activity Readiness Questionnaire

The Physical Activity Readiness Questionnaire is an internationally renowned preparticipation screening tool that was based on expert opinion (British Columbia Ministry of Health and the Multidisciplinary Board on Exercise) [100]. It intends to find whether the test participant should see a physician before beginning physical activity or a sport. Here, cardiovascular, balance, medical, emotional, and joint issues, which could hinder physical activity or sports activity, should be excluded. All questions should be answered with *no.* This questionnaire is used as an exclusion criterion in this study and is used in the telephone survey. If a question is answered with *yes,* the person is not included in the study.

### Psychological Stress Tests (T0 and T2)

#### Stress Induction Paradigms

As the study design aims to investigate acute stress effects at several time points (T0 and T2) by focusing on the change in saliva cortisol levels as a primary end point, 2 alternative paradigms for stress induction are required, which are described below. Thus, habituation and expectation effects can be reduced based on the first stress induction paradigm. These psychological stress tests will be conducted between 1:30 PM and 4 PM to control the circadian cortisol level. To eliminate possible contaminations of saliva, participants will be instructed to refrain from eating or drinking for 1 hour before the stress tests. In addition, participants will watch a relaxing movie for approximately 20 minutes to minimize baseline differences in cortisol concentrations.

#### Socially Evaluated Cold Pressure Test (T0)

Stress induction at T0 will be performed by means of the SECPT [49]. The participant will be asked to immerse their nondominant hand in a pool of cold water (0-4 °C) and to keep it there if possible. During this time, the participant will be observed by an experimenter, who will keep a neutral facial expression while taking notes on a clipboard. To further enhance the social evaluation, the face of the participant will be recorded with a mock camera. The participants will be encouraged to keep their hands immersed for a maximum of 3 minutes.

#### Psychosocial Stress Task for Scanner Environments (T2)

Stress induction at T2 will be performed using the compact version of the psychosocial stress task for scanner environments (ScanSTRESS) paradigm [50,52]. First, the participants will go through a control phase and then a stress phase (6 minutes each). During the stress phase, 2 types of cognitively challenging tasks will be processed with computer assistance (Neurobs Presentation software, Neurobehavioral Systems): mental rotation and arithmetic subtraction tasks. A preprogrammed algorithm adapts the task difficulty and speed to the participant´s performance, thus creating time pressure and forcing errors. The social-evaluative component is a jury (consisting of 2 test supervisors in white lab coats) that is transmitted via a live video stream to the screen of the participant during the task processing and thus continuously providing negative disapproving feedback. In case of too slow processing, as well as in the case of an erroneous answer, the jury can also project negative written feedback onto the respondent's screen via a buzzer (“Work faster!” and “Error!”). To further increase the stress level, the stress phase will be interrupted halfway through for verbal feedback. Here, the participant will be reminded that the data can only be used if maximum performance is achieved. Accordingly, the highest possible concentration is requested. During the control phase, the participants will only perform simple assignment tasks without time pressure and negative feedback.

#### Saliva Cortisol

To validate the effectiveness of the stress tests performed, 6 saliva samples will be collected throughout the experiment to detect the stress hormone cortisol. Commercially available Salivettes (Sarstedt) will be used for this purpose. All saliva samples will be stored at –20 °C and sent to the Institute of Biopsychology at the Technical University Dresden, Germany, for analysis. Salivary concentrations will be measured using commercially available chemiluminescence immunoassays with high sensitivity (IBL International).

### Emotion Induction and Regulation (T0 and T2)

To detect differences in the ability to regulate emotions, 2 different tests will be used in parallel to the stress tests.

#### Cognitive Emotion Regulation Task (T0)

The Cognitive Emotion Regulation Task (CERT) [101,102] comprises 3 task conditions and 2 image categories. In the first task condition, the participants are instructed to merely watch the presented stimuli and react naturally to them (*view condition*). In the second task condition, the participants are asked to cognitively modify the content of the presented image by assigning it a positive content (*cognitive reappraisal*) and thereby regulating their emotions. In the third task condition, participants are asked to indicate as fast as possible if the given mathematical equation is correct or incorrect via a button press (*distraction*). For this purpose, the participants are presented with both negative (first image category) and neutral images (second image category). The images are taken from the internationally standardized and evaluated databases *International Affective Picture System*) [103] and *Emotional Picture Set* [104]. After 1000 ms of stimulus presentation, the task instructions are presented for 1000 ms (transparent overlay). The image stimulus is then on display for another 5000 ms, followed by a 4000-ms rating phase where participants indicate their current affective state using the *Self-Assessment Manikin* scale for valence [105]. The interstimulus interval is 3000-5025 ms. In our study, the CERT comprises 75 trials of approximately 14.5 seconds each, 15 trials each for the following conditions: view_negative, view_neutral, distract_negative, distract_neutral, and reappraise_negative. In total, the CERT lasts approximately 19 minutes.

#### Script-Based Reappraisal Test (T2)

The Script-Based Reappraisal Test [106] is a computer-based behavioral experiment that assesses an individual’s ability to regulate emotions. In this experiment, text-based scripts will be presented that describe everyday situations and induce negative emotions (anger toward others, anger toward oneself, and fear). In this experiment, participants will work through several passages in which a script is first presented (20 seconds). Subsequently, participants will be instructed, depending on the passage, to reduce the emerging negative emotions within 1 minute by cognitive reassessment (reassessment passages) or to allow them to arise (control passages). At the end of each session, the participants will use *Self-Assessment Manikin* scales [105] to indicate their affective state in terms of valence and arousal (6 seconds) and type in their revaluation thoughts or negative thoughts (90 seconds). The Script-Based Reappraisal Test contains 12 revaluation sessions and 12 control sessions. At the beginning of the experiment, 2 sample runs will be performed, and the participants' questions on understanding will be answered.

### Peak Oxygen Uptake Determination (T1 and T3)

To determine the individual VO_2_peak as the primary end point and the training areas, participants will complete a stepwise incremental running test on a treadmill (Saturn, HP-Cosmos) until subjective exhaustion is reached or general indications for stopping an exercise test according to the American College of Sports Medicine guidelines are fulfilled [59]. Maximal oxygen uptake is reached if a plateau of VO_2_ is observed within the final 2 work rates of the stepwise incremental running test [59]. Here, we have untrained participants who may not be able to reach a plateau in VO_2_ uptake. Therefore, we will use VO_2_peak and take the highest value of VO_2_. To avoid outliers, there should be no high variance around this VO_2_ value within the 4 previously taken breaths. The exercise test includes a starting velocity of 4 km/hour, a duration of each step of 3 minutes, a pause time between 30 seconds and 60 seconds, and an increase in velocity by 1.5 km/hour from step to step. To determine the lactate threshold, 20 µL of whole blood will be collected from the fingertip with an end-to-end capillary (Sodium-Heparin, EKF-Diagnostics GmbH) at rest and between the stages. Heart rate (electrocardiogram), as well as oxygen uptake and carbon dioxide release (spiroergometry), will be continuously recorded. The latter will be used to estimate VO_2_peak for IG and CG at T1 and T3. VO_2_peak at T1 will be considered as innate aerobic capacity, and VO_2_peak of the IG at T3 will be considered as acquired aerobic capacity. Absolute and relative contraindications will be defined according to the American College of Sports Medicine guidelines [59].

### Infrared Thermography (T1 and T3)

Infrared thermography is a noninvasive, contact-free, nonradiating tool used to measure surface radiation temperature and patterns [107]. In this study, a high-resolution infrared camera (Jenoptik VarioCam; 480×640 pixels; focal plane array; spectral range 7.5-14 micrometers; accuracy ± 0.05 K; and set emissivity: 0.98) will be applied for the measurement of mean surface radiation temperature (*T*_sr_ [°C]) of the calves (C*T*_sr_). The infrared camera will be placed on a tripod behind the participant at the height of 85 cm. The distance between the camera and participant will be 2.30 m. The focus will be set perpendicular to the backside of the calves to capture the area from the popliteal space to the ankle. To stabilize the running position on the treadmill, a horizontal barrier made of elastic-plastic will be fixed behind the participant, and the foot position will be marked on the treadmill with adhesive tape. The infrared camera temperature scale will be set from 25 °C to 35 °C. An acclimatization period of 10 minutes will be implemented before the exercise test. The participants will only be allowed to wear ankle socks and shorts; should sleep and fast for 8 hours; should drink not more than 1.5 L of water; should be physically inactive; and should avoid cosmetics, showering, and sunbathing for at least 8 hours before the test.

We will perform 3 steps for thermogram analysis: (1) thermogram selection, (2) thermogram processing, and (3) analysis of the region of interest. We will process the image by coloring everything in black, except for the relevant area in the thermogram. This analysis procedure is in line with that of Ludwig et al [108]. The final regions of interests analysis will be performed with the software *Analysis of thermal images–TDM V2.0* (Optoprecision GmbH). The report of the entire infrared thermography procedure is in accordance with that of Moreira et al [109].

### Measurements of Blood Parameters (T1 and T3)

Before, immediately after, and 30 minutes and 60 minutes after the exercise testing, the median cubital vein will be punctured for venous blood sampling. In total, 100 mL of whole blood will be taken and further processed for the preparation of blood serum and plasma aliquots, depending on the subsequent analysis. Blood counts and physiological parameters will be assessed from the venous plasma or serum, respectively, at the defined time points. The following parameters will be included: creatine kinase, lactate dehydrogenase, hydroxybutyrate dehydrogenase, calcium, cholesterol, triglycerides, high-density lipoprotein-associated cholesterol, low-density lipoprotein-associated cholesterol, total leukocytes, erythrocytes, hemoglobin, hematocrit, mean corpuscular volume, mean corpuscular hemoglobin, platelets, neutrophils, eosinophils, basophils, monocytes, lymphocytes, and total protein. Blood plasma samples for molecular marker analysis will be aliquoted and stored at –80 °C before further processing. Venous blood will be used to determine and analyze venous cell-free DNA (concentration using quantitative real-time polymerase chain reaction [110,111]); non–disease-specific epigenetic patterns and sequence analysis using targeted next-generation sequencing [112]; endocannabinoids, catecholamines, and inflammatory lipids using mass spectrometry [113,114]; and further circulating signal transducers (extracellular vesicles and cytokines using multiplexed assays [115-117]). In parallel with venous blood sampling and additionally at each increment of the exercise test, capillary blood samples will be taken from the fingertip to determine capillary cell-free DNA concentrations.

### Lactate Threshold and Determination of the Training Ranges (T1 and T3)

On the basis of lactate kinetics during the incremental stepwise running tests, the individual anaerobic threshold (IAT) will be used to determine the intensity ranges (regeneration, light, moderate, and vigorous) for the training sessions. The IAT will be determined by means of minimum lactate equivalent +1.5 mmol/L [118,119]. Here, we will define 4 training intensities (regeneration: 50%-70%, light: 70%-85%, moderate: 85%-100%, and vigorous: 100%-110%), which are relatively related to the velocity at IAT (100%).

### Training Intervention

**Figure 3:**
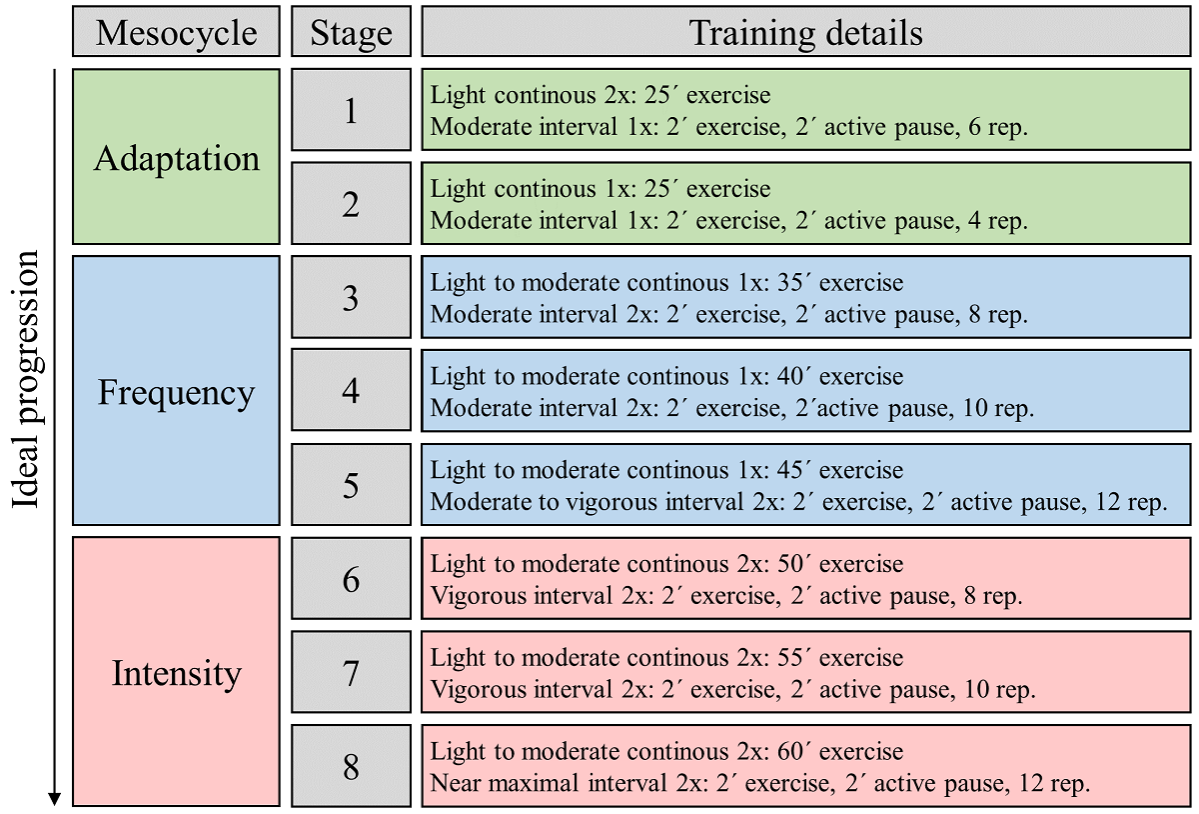
Ideal progression of training load from eight stages over the 8-week sport intervention period. Rep: repetitions.

After the baseline examination (T0 and T1), an 8-week sports intervention will be carried out for the IG. The CG will not participate in any training interventions during this period. The training contents are interval- and continuous endurance–oriented. The external load can be individually controlled and adjusted with the help of the IAT and the related relative training intensities. The intervention is divided into 3 mesocycles and 8 microcycles (=1 week): adaptation (2 weeks), frequency (3 weeks), and intensity (3 weeks; Figure 3). One box (Training details) corresponds to a fixed training week from Monday to Sunday. 5 minutes warm up and 5 minutes cool down should be included in every training session. The numbers 1x and 2x are the weekly frequencies of the training session.

**Figure 4:**
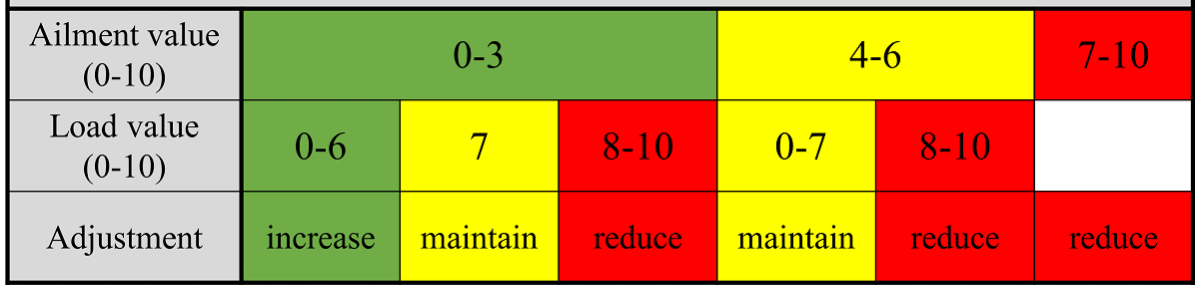
Individual training adjustment using the modified Borg scale [120] and the FITT-VP principle [43,45,59]. FITT-VP: Frequency, Intensity, Time, Type, Volume, Pattern, and Progression.

Here, a comprehensible protocol is used to adjust the training load using the principle of periodizing and cyclizing (frequency, intensity, time, type, volume, pattern, and progression; Figure 4) [43,45,59]. The gradual progression of the training load is based on weekly feedback of the participant via the Foster scale (0-10) [120]. The value for ailments has a higher priority than the load value. An optimal training load increase over 8 weeks of the training intervention is shown in Figure 3. After 8 weeks, each participant in the IG can end the training intervention on another stage based on the weekly feedback. In addition to the exercise training, the participants can conduct relaxation exercises in the form of stretching and mobilization exercises.

IG and CG participants will receive a smartwatch (M430, Polar Electro Oy) to record training times and monitor daily activities (eg, step count and sitting time). In addition, smartwatch data will be used to control for additional physical activity apart from the training intervention in the IG and physical activity, in general, in the CG. Furthermore, it will be used as a mechanism to control for the effect on primary outcomes by only receiving an intervention. The heart rate and distance of the training session will be recorded by the IG using the M430, which enables comparison of the data reported via the weekly feedback with smartwatch-recorded data. In the CG, the smartwatch is only used to record daily activity. To improve adherence of all participants, 15 smartwatches will be raffled among all participants who have completed the study.

### Web-Based Supervision

**Figure 5:**
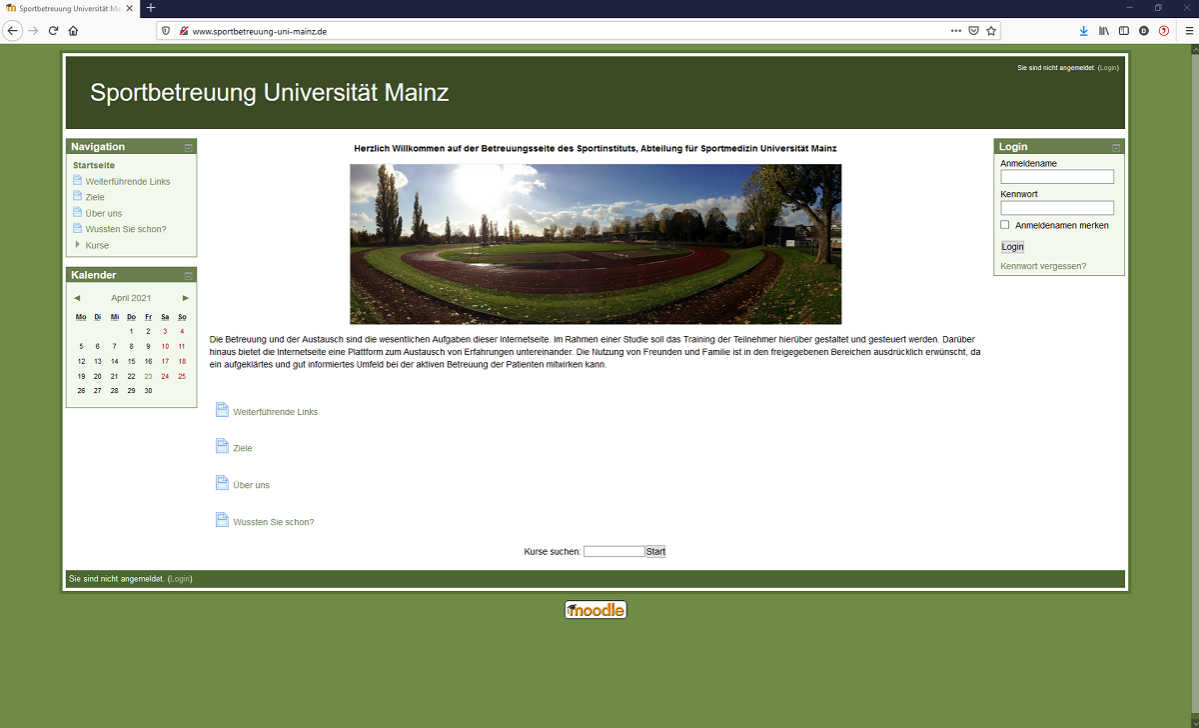
The home screen of the used website [121].

The training intervention described above will be accompanied and remotely supervised with the help of a web-based physical exercise training support via a website (Figure 5) [121]. In a personal appointment after the incremental stepwise running test, the participants in the IG will be introduced to the website and will receive comprehensive information about the training. External access to the website is not possible as only study participants can register. Both the introduction and the remote training supervision will be done by 1 person, which enables the coach to supervise several participants in their training process without any time constraints. Although there is no face-to-face supervision, based on the objective training session heart rate data and GPS data as well as the subjective feedback, adequate supervision can be guaranteed remotely. Participants have the possibility to get in direct contact with the coach via a message function, for example, in case of problems or uncertainties. The weekly training schedule is also transmitted in this manner. If a participant misses the feedback, a reminder is sent via email at the beginning of the following week. In a table that participants can download from the website, they will enter various medical and sports values that are therapeutically relevant after each training session. These data will be used for evaluation and for the training adjustment of the following week, as described in Figure 4. A follow-up is planned at 12 weeks (T4) and 24 weeks (T5) after completion of T3, and participants will be asked via email to complete the web-based questionnaires used at T2.

### Data Management and Security

All data will be recorded and stored in the Department of Sports Medicine, Prevention, and Rehabilitation (Mainz, Germany), and access will be restricted to authorized personnel. Confidential information will be stored safely in the trial files and will not be shared with any other third institution or entity except in response to a legal requirement. Electronic data will be stored on password-protected computers in a restricted-access building. All data will be pseudonymized and submitted to the evaluation team for analysis. A data manager from each department is nominated, who will be responsible for data management and processing. The data and safety management team will be formed by the members of the project. Only the data manager will have access to all personal data. Trial documentation and data will be archived for 10 years after completion of the trial. All dropouts and the reason for dropping out of the study will be reported. Any harm or unintended events during the examinations will be recorded and reported to the Landesärztekammer (State Medical Association) Rhineland-Palatinate. The results will be publicly available in open-access journals and presented via contributions at conferences and congresses according to good scientific practice.

### Statistical Methods

The power calculation was based on the analyses of the primary outcomes, that is, aerobic capacity (VO_2_peak) and experimental stress reactivity (saliva cortisol). On the basis of the results of Bacon et al [122], the sample size was determined a priori via power analysis (G*Power V5), yielding a minimum sample size of 67 for a medium-to-large effect size of *f(V)*=0.35. An alpha error set to .05 and statistical power of 0.80 were assumed for a multivariate analysis of variance (MANOVA) with 2 repeated measurements, 2 groups, and interaction effects. Descriptive statistics will be used for all outcome measures at each time point to give an overview of all results. First, MANOVA will be performed with the main primary outcomes as dependent variables, that is, aerobic capacity (VO_2_peak) and experimental stress reactivity (saliva cortisol) to maximize the power of the analyses and to be able to investigate the main and interaction effects of time (baseline [T0, T1] vs post [T2, T3]) and group (IG vs CG). Groups should be equal at preintervention (T0 and T1). A time effect should be observed for the IG. Furthermore, we expect a significant time×group interaction as the IG should react differently over time, with a higher increase in aerobic capacity (VO_2_peak). Significant effects of the MANOVA will be followed up by posthoc analyses of variance. As a secondary aim of our study, we aim to identify variables (eg, emotion regulation and biological metrics) that mediate the relationship between physical exercise and resilience outcomes. To that end, we plan to calculate multiple regression analyses that include potential mediators. Principal component analyses regarding potential mediating variables will precede the mediator analyses to avoid redundancy in our regression models. This will be statistically detailed with posthoc tests and corrections for multiple comparisons (Bonferroni-Holm correction [123]). Missing data will be omitted from the data set.

## Results

The study was registered in October 2019 (DRKS00018078). Enrollment began in September 2019 and was paused from April 2020 until submission of this study protocol because of the COVID-19 pandemic. Of the 161 interested people who have contacted us so far, 26.7% (43/161) fulfilled the inclusion criteria. Among the 55% (17/31) participants in the IG and 45% (14/31) participants in the CG, who completed the study (N=31), no serious adverse incidents were reported. Within the program, 9% (4/43) of participants (2/17, 12% in the IG and 2/14, 14% in the CG) withdrew because of individual reasons (dropout rate 11.4%). Approximately 19% (8/43) have not yet participated in the program because of the COVID-19 pandemic. Although the COVID-19 pandemic does not affect the web-based exercise training approach, the study had to be paused in April 2020, as it was not possible to determine the primary and secondary end points (VO_2_peak and cortisol levels) because of the lockdown of the laboratory facilities. Further study recruitment will resume when COVID-19 restrictions are completely lifted. On the basis of the experiences from the first recruitment period, the study is expected to continue for another 6 months to complete the intended sample size.

## Discussion

### Benefits of the Study

Evidence that exercise can lead to various benefits in mental health and resilience is increasing [16,124-129]. This positive impact was achieved in supervised as well as unsupervised exercise programs. Here, we implement a study design that addresses the link between aerobic capacity and resilience from a multifactorial perspective to understand why some people are more resilient to stress than others. To follow this question, we will investigate the association between innate and trained aerobic capacities in the form of VO_2_peak of healthy, physically inactive adults and their resilience expressed primarily by salivary cortisol level changes and emotion regulation ability in response to different stress paradigms. We hypothesize that (1) participants with a high innate aerobic capacity will show a higher resilience to stress compared with participants with low aerobic capacity. Furthermore, we hypothesize that (2) 8 weeks of individually structured interval-type and continuous endurance exercise training remotely supervised by a web-based approach will improve the aerobic capacity of healthy inactive adults and that (3) the change from sedentary to an active lifestyle will improve resilience. In this study setting, we will be able to exploratively examine the molecular link between the effects of acute and chronic physical exercise and improved resilience to stress.

### Strengths and Limitations

The strengths of our web-based, supervised physical exercise training approach with a focus on increasing VO_2_peak in healthy, physically inactive adults are as follows: (1) participants are not location dependent for the training sessions; (2) individualized, gradual, and timely adjustment of training content based on the individual’s feedback reduces the risk of injury, avoids overtraining, and avoids ineffective training; and (3) only 1 sports therapist or trainer is needed for training management of several training groups or trainees, which obviously has economic advantages and reduces bias from different sports therapists. However, web-based training support also has disadvantages compared with presence (face-to-face) interventions. Notably, it requires access to the internet, which limits the study population. Furthermore, we are dependent on the correct and complete documentation of the training sessions by the participants. For an objective way to control reports on the training and to remotely supervise the participants in the IG, we included a smartwatch (Polar M430) that records the heart rate and distance of the individual training sessions. These are given to both the IG and CG participants to exclude an effect on the study outcome only by wearing the smartwatch. Furthermore, group or partner training can bring motivational and social benefits [130-132], which is not directly provided in our web-based physical exercise training support. This may reduce adherence of the participants to the study and, additionally, exclude the social aspect of the effect of an active lifestyle on resilience. Therefore, communication via the web-based platform is intended to be frequent and without any restraints toward the trainer. Finally, inexperience, fear, and the risk of injury can occur during an unaccustomed and intensive training load, which could be reduced or not arise at all through presence support. To counteract this, we have implemented a 2-week adaptation phase in our training intervention that will offer an easy training start and reduce the indicated risks. In addition, we address the individual needs of the participants based on their weekly feedback, so overload is unlikely to occur during the intervention. Adhering to the described improvements in web-based physical exercise interventions will offer the most individual and effective training support with a simultaneously reduced workload for the coach. With this, our research group has already successfully conducted similar studies using the internet for training interventions in patients with Barrett carcinoma, nonalcoholic fatty liver disease, depression, cystic fibrosis, and systemic lupus erythematosus [40-45].

Here, we selected an exercise intervention period of 8 weeks using a combination of interval-type training and continuous endurance exercise to efficiently enhance aerobic capacity in a short period. Interval training improves VO_2_peak more strongly in comparison with continuous exercise training [8]. Moreover, psychological benefits in attention, complex attention, and executive function were shown in high-intensity interval training compared with those in low-intensity training [133-135]. However, to counteract overtraining and risk of injury and to promote adherence, we complement interval-type training with endurance exercise sessions. A number of high-intensity interval training and moderate-intensity intervention studies with physically inactive participants decided for a comparable exercise intervention time leading to significant increases in VO_2_peak of 3%-19% [9-11]. Although a longer intervention period generally leads to higher increases in maximal oxygen uptake [122], it has been reported that enjoyment of interval training decreases with time [10]. Therefore, we considered a study design that used mixed model exercise sessions with a short intervention time of 8 weeks as efficient to enhance aerobic capacity in physically inactive adults and as suitable to analyze the impact on resilience.

Repeated stress induction and the estimation of resilience factors in a test-retest setting, as chosen in our study, are obstacles to resilience research. It was shown that for psychological stress tests, a test-retest design could lead to a learning effect, resulting in lower stress response [136,137]. Here, we use 2 different stress tests for T0 and T2 to reduce habituation to the applied stressor. By randomly assigning participants to the 2 groups, we intend to obtain the best estimate of the intervention effect on stress responses. However, differences in stress parameters at T2 might not exclusively be attributed to the intervention, as possible placebo effects (ie, simply receiving an intervention) or mediators may need to be considered. Previous studies analyzed similar between-subject comparisons, with less stress reactivity during the Trier Social Stress Test in trained men compared with untrained men [138]. In addition, we account for preintervention differences in stress reactivity and recovery by applying the SECPT at T0. However, as measures from both stress tests are not directly comparable, the experimental design does not permit within-subject analyses. To assess differences in individual changes within the IG, future studies may construct and apply parallel versions of a stress test pre and postintervention.

Several psychological interventions have been similarly used to increase resilience [139], and it might have been interesting to have a sport exercise group; a psychological exercise group that uses a psychoeducational element, such as cognitive restructuring; a mixed model group (sport and psychological); and a CG to obtain the influence of different types of interventions and their combinations on resilience to stress exposure. In our study, we focus on the physiological aspects of increasing VO_2_peak and its effect on resilience only. However, the identified relationships between molecular markers and exercise-induced resilience parameters in our setting may be further elucidated in mixed model interventions in follow-up studies.

We cannot exclude a selection bias so that persons who are generally interested in (1) psychological questions, (2) physical exercise, or (3) web-based services are rather addressed by the study announcement. Furthermore, because of a high time commitment for presence tests (2 measurement time points before and after the intervention), (4) people who live close to the survey site are more likely to participate and complete the study. On the basis of the World Health Organization guidelines on physical activity and sedentary behavior, we included individuals who were physically active for <150 minutes/week [140]. These individuals are described as insufficiently active, although this definition is very strict. Possibly, a cutoff point of physical activity <90 minutes/week would obtain a greater effect on the primary outcomes. Here, we decided in favor of the adherence to the study considering the training times that had to be completed.

Resilience is a dynamic and variable state that can be represented in a multifactorial manner. So far, individual instruments have failed to fully capture the variance of resilience [17,141,142]. For this reason, our study uses a wide variety of methods (physiological [blood markers, saliva markers, and hair] and psychological [questionnaires, stress tests, and emotion regulation]) to have a large number of independent predictors to clarify the variance in resilience to the greatest degree of accuracy. Owing to the high number of methods and the collected variables, spurious correlations or pseudoassociations may occur [143], especially when exploratory analysis is performed beyond hypothesis testing. The validity of these findings may be estimated in future studies.

### Conclusions

This study will allow us to investigate whether an increase in physical activity with a simultaneous improvement in aerobic capacity is associated with an increase in resilience and whether this effect is reflected in circulating molecular marker levels. These findings will help to reveal novel characteristics of human resilience and may offer novel approaches for the prevention and therapy of mental disorders by exercise prescription.

## Appendix

Multimedia Appendix 1 CONSORT-eHEALTH checklist (V 1.6.1).

Multimedia Appendix 2 Flyer announcement for the study.

## Abbreviations

ADS: Allgemeine Depressionsskala
ADS-L: Allgemeine Depressionsskala–Long Version
CERT: Cognitive Emotion Regulation Task
CG: control group
CTS: Childhood Trauma Screener
HPA: hypothalamic-pituitary-adrenal
IAT: individual anaerobe threshold
IG: intervention group
MANOVA: multivariate analysis of variance
MDBF: Mehrdimensionaler Befindlichkeitsfragebogen
PSS: Perceived Stress Scale
SCS: Self-compassion Scale
SECPT: socially evaluated cold pressor test
SPIRIT: Standard Protocol Items: Recommendations for Interventional Trials
TICS-SSCS: Trier Inventory for the assessment of Chronic Stress–Screening Scale for Chronic Stress
VO_2_peak: peak oxygen uptake
